# CryoEM structure of a post-assembly MS-ring reveals plasticity in stoichiometry and conformation

**DOI:** 10.1371/journal.pone.0285343

**Published:** 2023-05-19

**Authors:** Prashant K. Singh, Gary Cecchini, Terunaga Nakagawa, T. M. Iverson

**Affiliations:** 1 Department of Pharmacology, Vanderbilt University, Nashville, TN, United States of America; 2 Molecular Biology Division, San Francisco VA Health Care System, San Francisco, CA, United States of America; 3 Department of Biochemistry & Biophysics, University of California, San Francisco, CA, United States of America; 4 Department of Molecular Physiology and Biophysics, Vanderbilt University, School of Medicine, Nashville, TN, United States of America; 5 Center for Structural Biology, Vanderbilt University, Nashville, TN, United States of America; 6 Department of Biochemistry, Vanderbilt University, Nashville, TN, United States of America; 7 Vanderbilt Institute of Chemical Biology, Vanderbilt University, Nashville, TN, United States of America; University of Nova Gorica, SLOVENIA

## Abstract

The flagellar motor supports bacterial chemotaxis, a process that allows bacteria to move in response to their environment. A central feature of this motor is the MS-ring, which is composed entirely of repeats of the FliF subunit. This MS-ring is critical for the assembly and stability of the flagellar switch and the entire flagellum. Despite multiple independent cryoEM structures of the MS-ring, there remains a debate about the stoichiometry and organization of the ring-building motifs (RBMs). Here, we report the cryoEM structure of a *Salmonella* MS-ring that was purified from the assembled flagellar switch complex (MSC-ring). We term this the ‘post-assembly’ state. Using 2D class averages, we show that under these conditions, the post-assembly MS-ring can contain 32, 33, or 34 FliF subunits, with 33 being the most common. RBM3 has a single location with C32, C33, or C34 symmetry. RBM2 is found in two locations with RBM2_inner_ having C21 or C22 symmetry and an RBM2_outer_-RBM1 having C11 symmetry. Comparison to previously reported structures identifies several differences. Most strikingly, we find that the membrane domain forms 11 regions of discrete density at the base of the structure rather than a contiguous ring, although density could not be unambiguously interpreted. We further find density in some previously unresolved areas, and we assigned amino acids to those regions. Finally, we find differences in interdomain angles in RBM3 that affect the diameter of the ring. Together, these investigations support a model of the flagellum with structural plasticity, which may be important for flagellar assembly and function.

## Introduction

Chemotaxis allows bacteria to follow a gradient of nutrients, signaling molecules, or other environmental factors [1]. Chemotaxis also enhances infectivity [2] and promotes bacterial pathogenesis [3] by influencing quorum sensing [4,5], imparting the motility required to reach a preferred site of infection, promoting adherence to the host cells [6], and triggering biofilm formation [7,8].

**Fig 1:**
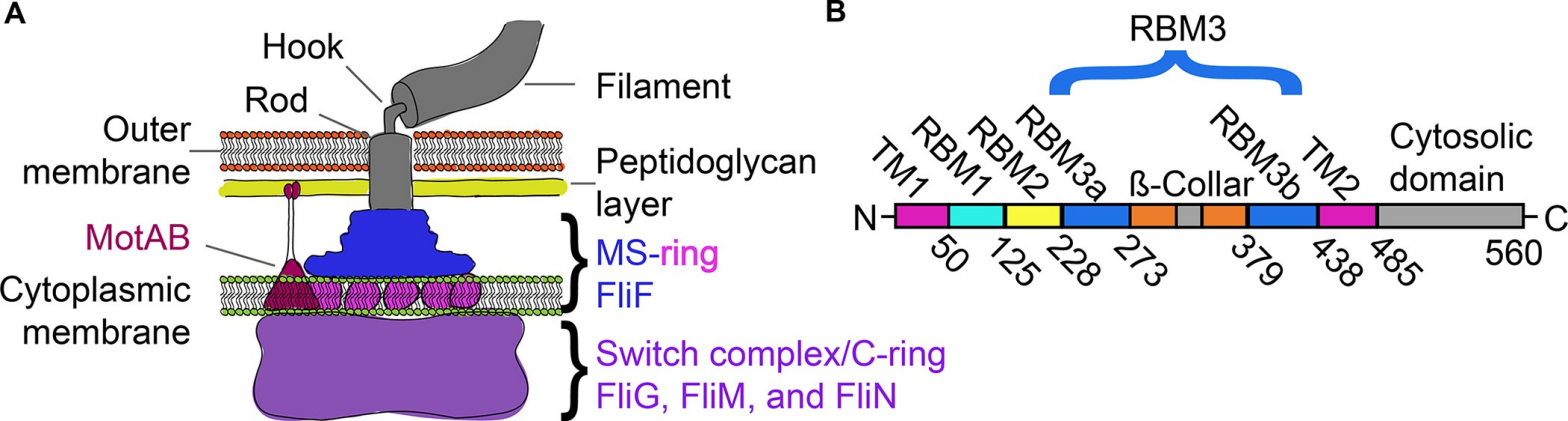
Overview of the bacterial flagellar motor complex and the MS-ring. **(A)** Schematic of bacterium flagellar motor. The soluble region of the MS-ring, sometimes called the S-ring, is shown in blue while the membrane-spanning region of the MS-ring, sometimes called the M-ring, is shown in magenta. Both the M-ring and S-ring are formed from FliF subunits. The switch complex, or C-ring, is shown in purple and contains FliG, FliM and FliN. **(B)** Linear schematic of the FliF protein sequence with the transmembrane regions colored magenta, RBM1 colored cyan, RBM2 colored yellow, RBM3a and RBM3b colored blue, the β-collar regions colored orange, and the cytosolic regions and linkers colored grey.

A biological structure central to bacterial chemotaxis is the flagellum. Low-resolution structures identified that the flagellum comprises various rings [9,10], which is consistent with a role as a rotary motor. Indeed, non-flagellated variants of *Salmonella* and a non-chemotactic mutant of *Heliobacter pylori*, have a reduced rate of infection [3,11]. Both the assembly and the rotation of bacterial flagellum depend upon the MS-ring of the flagellar motor. One specific structure that requires the MS-ring is termed the switch complex, which is also known as the C-ring. This switch complex is built from many copies of FliG, FliM, and FliN **(Fig 1A)** and controls the direction of rotation of the flagellum. The importance of the MS-ring for flagellar assembly and chemotaxis has led to intense interest in its composition and architecture.

The MS-ring is composed exclusively of multiple copies of the membrane-spanning FliF subunit [12]. It is therefore not surprising that mutations in *Salmonella enterica* FliF can lead to impairment of both motility and chemotaxis [13,14]. The polypeptide chain of FliF organizes in a modular way (**Fig 1B)**. Starting at the N-terminus, each FliF protomer begins with a predicted transmembrane helix, TM1, [15,16] followed by a set of domains termed ring-building motifs (RBMs). From the N-terminus, these include RBM1, RBM2, and RBM3, with RBM3 further divided into RBM3a, a ß-collar, and RBM3b. Although RBM3a and RBM3b are not contiguous in the primary sequence, they form a single domain in the overall structure [17]. Following the three ring-building motifs, a second predicted transmembrane helix, TM2, precedes a C-terminal cytosolic domain.

At present, there are both high-resolution cryoEM structures of the MS-ring from *S*. *enterica* [17–21] as well as low-resolution structures [9,22]. Of the structures where a model could be built, one is from FliF expressed in isolation in *E*. *coli* [17]. This structure may represent the state of the MS-ring prior to the assembly of the flagellum or the switch complex. There is one structure of the MS-ring determined from assemblies that formed following coexpression of FliF and FliG [20] and may represent an intermediate of the process of switch assembly. Finally, there are three structures of the MS-ring in the context of other regions of the *S*. *enterica* flagellum [18,19,21].

Each of these MS-ring structures shows a similar fold for the RBM2 and RBM3 domains [17–20]. In addition, the assignment of FliF protomers to each of these structures [17–20] shows different numbers of interpretable RBM domains, *i*.*e*., one FliF subunit might contain three observable RBMs, another might only contain an observable RBM2 and RBM3, and yet another might only have observable RBM3. Presumably, the remaining regions of the FliF subunit are unfolded, disordered, or in conformations that were not identified using cryoEM. This results in a difference in the number of RBM1, RBM2, and RBM3 domains assigned to the MS-ring and a symmetry mismatch.

Although many parts of these structures agree, there are also aspects of each of these structures that differ. The first difference, and one of intense debate, is the number of FliF subunits in the MS-ring. The first reported structure [17] identified that the MS-ring could contain anywhere between 32 and 36 FliF subunits. Subsequent reports, however, were consistent with a homogeneous MS-ring containing 34 subunits [18–21]. One interpretation of these stoichiometric differences is that there is more than one biologically relevant state of the MS-ring, and the different preparation methods captured a predominant state. On some level, variation in MS-ring stoichiometry is biologically plausible because the number of subunits in the flagellum may be able to change spontaneously in response to load [23–25]. An alternative interpretation is that 34-mer MS-rings represent the correct architecture, and the other stoichiometries are artifacts arising from proteolytic truncations at the FliF C-terminus or particle misalignment [19].

The second difference in these structures is the variability in the number and position of folded RBM2 domains. All structures have an RBM2 ring positioned just beneath the RBM3 ring, with this ring termed RBM2_inner_. The various reports identify between 21–23 folded RBM2_inner_ domains, with all reports agreeing that there is more than one stoichiometry [17,19,20]. The RBM2 can also be found in a second position on the periphery of the RBM2_inner_ ring, with this second position termed RBM2_outer_. RBM2_outer_ interacts with RBM1 in discrete densities and does not form a contiguous ring. However, the stoichiometry again differed, with either nine RBM2_outer_-RBM1 densities exhibiting 3-fold symmetry [17] or 11 RBM2_outer_-RBM1 densities exhibiting 11-fold symmetry [20]. Because the contacts between RBM2_outer_ and RBM1 are analogous to those observed in a crystal structure of the *Aquifex aeolicus* RBM2-RBM1 [20], this interdomain interaction is likely to be biologically relevant.

A third major difference in the reported structures of the MS-ring is in the observation, stoichiometry, and positioning of RBM1. RBM1 is sometimes assigned as an isolated globular domain positioned below RBM2_outer_ [17]. When assigned to this position, there are nine RBM1 domains with C3 symmetry. RBM1 has also been assigned as a tight heterodimer with RBM2_outer_ [20]_._ When in this position, there are 11 RBM1 domains with C11 symmetry. Both assignments show discrete regions of density rather than a connected ring [17,20]. Notably, the structures that do contain RBM1 [17,20] have a substantially lower resolution in this region of the MS-ring. A crystal structure of the *A*. *aeolicus* RBM1 [20] shows that it folds into a small globular domain that is dominated by two helices; the density in each of the cryoEM structures is consistent with this fold [17,20].

All of these controversies surround the three RBMs. This is because, to date, all of the high-resolution density [17,20] of the MS-ring has been associated with RBM3, RBM2, and RBM1. In contrast, the density corresponding to the membrane-spanning region of FliF has only been observed as a contiguous ring at the base of the structure with uninterpretable density.

To inform on the controversies surrounding the MS-ring architecture, we prepared samples in a different way. We leveraged the fact that the MS-ring is used to template assembly of the flagellar switch complex (FliGMN or C-ring) and we coexpressed *Salmonella enterica* FliF with FliG, FliM, and FliN. We then extracted these MS-rings away from the assembled switch complex, terming this a ‘post-assembly’ complex. FliF prepared in this way was highly stable, showed no proteolysis, and unlike the samples used in previous MS-ring structures [17,20] did not require crosslinking. In these post-assembly MS-rings, we find structural plasticity in both stoichiometry and domain placement. Further, we observe that the membrane spanning regions may form discrete domains with C11 symmetry.

## Materials and methods

### Constructs

Plasmid pKLR3-FliFGMN [26] was a generous gift from Dr. Michael Eisenbach, who originally received it from Dr. Shahid Khan.

### Protein expression and purification

*S*. *typhimurium* FliFGMN was expressed in *E*. *coli* BL21-Gold cells in LB supplemented with 0.034 mg/ml chloramphenicol. At an OD_600_ of 0.6, expression was induced with 1 mM isopropyl β-D-1-thiogalactopyranoside (IPTG). Following induction, cells were grown at 37°C for 18 hours with shaking, then harvested by centrifugation at 6,750 x *g* at 4°C. The cells were resuspended in lysis buffer (50 mM Tris-HCl pH 7.5, 100 mM NaCl) with one protease inhibitor tablet (ThermoScientific Cat# A32965) for every 50 ml of buffer. Cells were lysed in a microfluidizer (LM20, Microfluidics) at 20,000 psi. The lysed cells were spun at 17,000 x *g* for 30 min to remove cellular debris and then the supernatant at 125,000 x *g* for 45 min to separate the membranes. These membranes contain the flagellar switch complex comprising both the MS- and the C-rings. MS-rings were extracted from the switch complex in solubilization buffer (50 mM Tris-HCl pH 7.5, 100 mM NaCl, 2% Ana-grade Lauryldimethylamine-N-Oxide, LDAO (Anatrace, D360)) with protease inhibitors and stirring for 1 hour. The solution was then centrifuged at 125,000 x *g* for 45 min to separate out the cell membranes (pellets) from the solubilized protein (supernatant) and the supernatant was collected for further purification.

In the first step of purification, the supernatant was passed through two ion exchange columns stacked together in sequence: a HiTrap SP cation exchange column (Cytiva, 17115201) and a HiTrap Q anion exchange column (Cytiva, 17115301). After binding, the cation exchange column was removed and the anion exchange column was washed with 10 to 15 column volumes of Buffer A (50 mM Tris-HCl pH 7.5, 100 mM NaCl, 0.1% Ana-grade LDAO). MS-rings were eluted with a linear gradient of 5%– 50% of 1M NaCl with protein eluting at 23% of the elution buffer (50 mM Tris-HCl pH 7.5, 1 M NaCl, 0.1% Ana-grade LDAO). The fractions containing isolated MS-rings were identified with negative stain electron microscopy. For negative stain, 5 μl of MS-rings at a concentration of approximately 50 μg/ml were applied to glow-discharged carbon-coated copper grids and incubated for 3 min. The grids were then washed twice in deionized water and incubated with 0.7% w/v Uranyl Formate for 1 min. The fractions containing FliF rings were pooled and further purified using a Superdex 200 increase 10/300 GL column (Cytiva Life Sciences) equilibrated with Buffer A. Protein was estimated to be >85% pure via SDS-PAGE.

### Cryo-EM sample preparation and imaging

A 400 mesh R1.2/1.3 Au Quantifoil grid coated with graphene oxide substrate (Electron Microscopy Sciences) was glow discharged for 5 sec. Purified MS-rings (2 μl of 0.5mg/ml) were added to each grid at 4°C at 100% humidity. After 30 sec of incubation, blotting was performed for 12 sec. The grid was then plunged into liquid ethane using a Vitrobot Mark IV system (Thermo Fisher). Data were collected at the Center for Structural Biology CryoEM Facility at Vanderbilt University using a 300 keV Titan Krios G4 microscope with a Falcon-III direct electron detector (Thermo Fisher). Detailed data collection statistics are provided in **Table 1**.

**Table 1:** Cryo-EM data collection, refinement and validation statistics.

	FliF RBM3 (EMDB-29425) (PDB:8FTF)	FliF RBM2 (EMDB-29424) (PDB:8FTE)
**Data Collection**		
Microscope	Thermo Fisher FEI Titan G4	Thermo Fisher FEI Titan G4
Detector	Falcon-III DED	Falcon-III DED
Magnification	120,000x	120,000x
Voltage (kV)	300	300
Electron exposure (e^-^/Å^2^)	50	50
Defocus range (μm)	-0.8 to -2.2	-0.8 to -2.2
Pixel size (Å)	0.6811	0.6811
Total micrographs taken	5,597	11,627
**Data Processing**		
Micrographs used for processing	5,597	10,707
Symmetry imposed	C33	C22
Initial particle images	336,352	173,942
Final particle images	32,042	100,096
Map resolution (Å)	2.91	3.8
FSC threshold	0.143	0.143
**Refinement**		
Initial model used (PDB)	PDB: 6SCN–RBM3	PDB: 6SD5
Model resolution (Å)	2.25 (masked), 3.0 (unmasked)	3.7 (masked), 3.8 (unmasked)
FSC threshold	0.143	0.143
Model resolution range (Å)	100–2.25	100–3.7
Model composition		
Total atoms	1285	667
Water	0	0
Protein residues	165	93
Bonds (RMSD)		
Bond lengths (Å) (# > 4σ)	0.002	0.003
Bond angles (°)(# > 4σ)	0.559	0.621
Validation		
MolProbity score	1.14	2.06
Clash score	3.53	3.69
Rotamers outliers (%)	0	7.9
Ramachandran plot		
Favored (%)	98	96.63
Allowed (%)	2	3.37
Disallowed (%)	0	0

We collected 5,597 un-tilted movies and 6,030 tilted movies bringing the total number of movies to 11,627 (**S1 Fig**). All movies were first motion-corrected using patch motion-based correction in cryoSPARC [27]; the last five frames from each movie were removed due to significant motion. Contrast Transfer Function (CTF) was estimated using Patch CTF Estimation in cryoSPARC [27]. After CTF was estimated, we removed micrographs with poor ice quality, resulting in 10,707 total micrographs. We followed two separate strategies to obtain the highest resolution structure of FliF assemblies with different stoichiometries, described below. For the 33-mer RBM3 map, we divided these into two groups, termed group “A” and “B” in **S1–S3 Figs**. Group “A” contained only the un-tilted micrographs (5,597 movies) while group “B” contained all 10,707 micrographs. For the remaining maps, including the 32-mer RBM3, 34-mer RBM3, 21-mer RBM2, and 22-mer RBM2, we used all micrographs (10,707 movies).

### Cryo-EM data processing for the MS-ring with C11 symmetry

We first built training models for particle picking using 1,000 un-tilted micrographs, which picked 12,065 particles (**S1 Fig**). This procedure resulted in two training models, which were top- and side-view specific. The models were used on the remaining micrographs using Topaz [28], a neural network-based particle-picking tool. This procedure picked 336,352 particles from the 5,597 un-tilted micrographs. Particles within 50 Å of each other were removed using star.py script from pyem [29], reducing the number of particles to 279,363. We classified these particles into 150 classes. After 2D classification, the best 21 class averages were selected (108,257 particles total). To separate different conformations and/or stoichiometries we performed heterogeneous refinement (3D classification) into four classes (**S2 Fig**). This used a 50 Å low pass filtered EM map of the MS-ring (EMD entry 10143 [17]) as a reference. The largest of the four classes contained 50,679 particles and was refined with C11 symmetry. This is the first time that symmetry was applied to the model. We then imposed C11 symmetry and performed heterogeneous refinement. The class with the best resolution and highest number of particles (33,903 particles) was subjected to another round of homogeneous refinement to give a map at about 3.7 Å resolution.

### Cryo-EM data processing for the 33-mer RBM3

On the above set of 50,679 particles that was a resultant of heterogeneous refinement, we performed C33 symmetry imposed heterogeneous refinement into two subclasses (**S2 Fig**). The best class contained 39,178 particles. The C33 particles were passed through the built-in duplicate particle removal tool in cryoSPARC [27] to remove 7,136 particles that were within 20 Å radius of another particle. The second round of refinement was performed on these 32,042 particles, followed by non-uniform refinement [30]. The resultant map corresponded to residues 228–438, exhibited C33 symmetry, and had a resolution of 2.9 Å. Local resolution estimation for this map ranged from 2.9 Å in the ß-collar region to 3.2 Å in the outermost region.

### Cryo-EM data processing for 32-mer and 34-mer RBM3

To develop models with C32 and C34 symmetry, we included the tilted micrographs in the data processing, which resulted in 193,430 additional particles (**S3 Fig**). The 2D class averages from the above C11 model were used as a template for particle picking. After the 2D classification, we separated the *en-face* views containing clear 32-mers and 34-mers into different starting classes. Because we are not able to distinguish between stoichiometries in the side views, we added all side views to each of these classes. This resulted in 46,160 particles for C32 and 52,778 particles for C34. We performed focused heterogeneous refinement on both C32 and C34 sets. The C32 and C34 maps with the best resolution and the greatest number of particles were selected for further refinement. A homogeneous refinement resulted in a map of RBM3 that extended to 4.2 Å (C32) and 3.9 Å (C34) (**S3 Fig**).

### Cryo-EM data processing for 21-mer and 22-mer RBM2

For the RBM2 ring, we began with heterogeneous refinement on all good particles irrespective of the oligomerization *i*.*e*., C32, C33, and C34 particles were all pooled together (173,942 particles) (**S3 Fig**). We then built a mask for the RBM2 ring region to use for local refinement [27]. Using this mask, we performed local refinements with imposed symmetry of C21 and C22 to obtain two initial models for further refinement. We then performed heterogeneous refinement on both models with imposed symmetry of C21 and C22 separately. Using the class with the best resolution and the greatest number of particles in both models, we performed a final local refinement which resulted in two separate models exhibiting 21-fold symmetry (90,962 particles) at 4.4 Å and 22-fold symmetry (100,096 particles) at 3.8 Å.

### Model building and refinement

Phenix Resolve CryoEM and DeepEMhancer [31] were used to improve the overall cryoEM map. The density was interpreted in Coot [32]. To build a model into the density, we docked the available RBM3 structure (PDB entry 6SCN [17]) and adjusted this model to match our density. The model was improved in Coot [32] using the 2.9 Å resolution map with C33 symmetry. Real-space refinement was performed in Phenix [33]. A similar model building procedure was performed on the 3.8 Å RBM2 map with C22 symmetry applied. A model was not built for RBM2 with C21 symmetry. The refinement statistics are provided in Table 1. The local resolution was estimated using CryoSPARC [27]. Figures including local resolution maps were prepared in ChimeraX [34]. The RMS deviation values were calculated in Coot [32]. The software for refinement and processing was administered by SBGrid [35].

### Results

#### Pre-symmetrized 2D classes suggest that the MS-ring can access multiple stoichiometries

**Fig 2:**
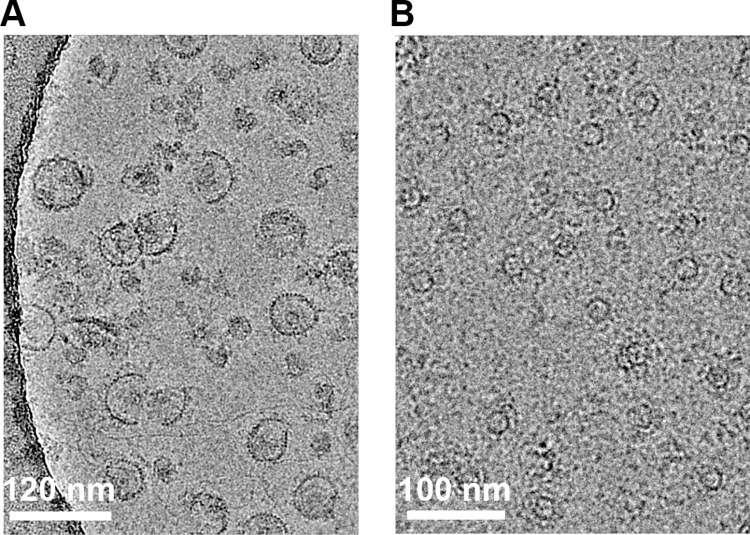
Representative cryoEM micrographs of samples used in this study. **(A)** Image of the assembled switch complex from which the MS-ring was extracted. The scale bar is 120 nm **(B)** Image of extracted MS-rings. The scale bar is 100 nm.

Given the present controversies on the structure of the MS-ring, we prepared this complex via a different method. Here, we coexpressed FliF with the flagellar switch (FliG, FliM, and FliN) to assemble the MS-ring and the C-ring (**Fig 2A**). We then extracted the MS-ring (**Fig 2B**). These MS-rings contained full-length FliF, remained stably assembled through this process, and showed no detectable proteolysis by SDS-PAGE (**S1A Fig**). Because these MS-rings are purified from a larger complex, we can be confident that they can support flagellar assembly. We used this sample to prepare grids for cryoEM.

**Fig 3:**
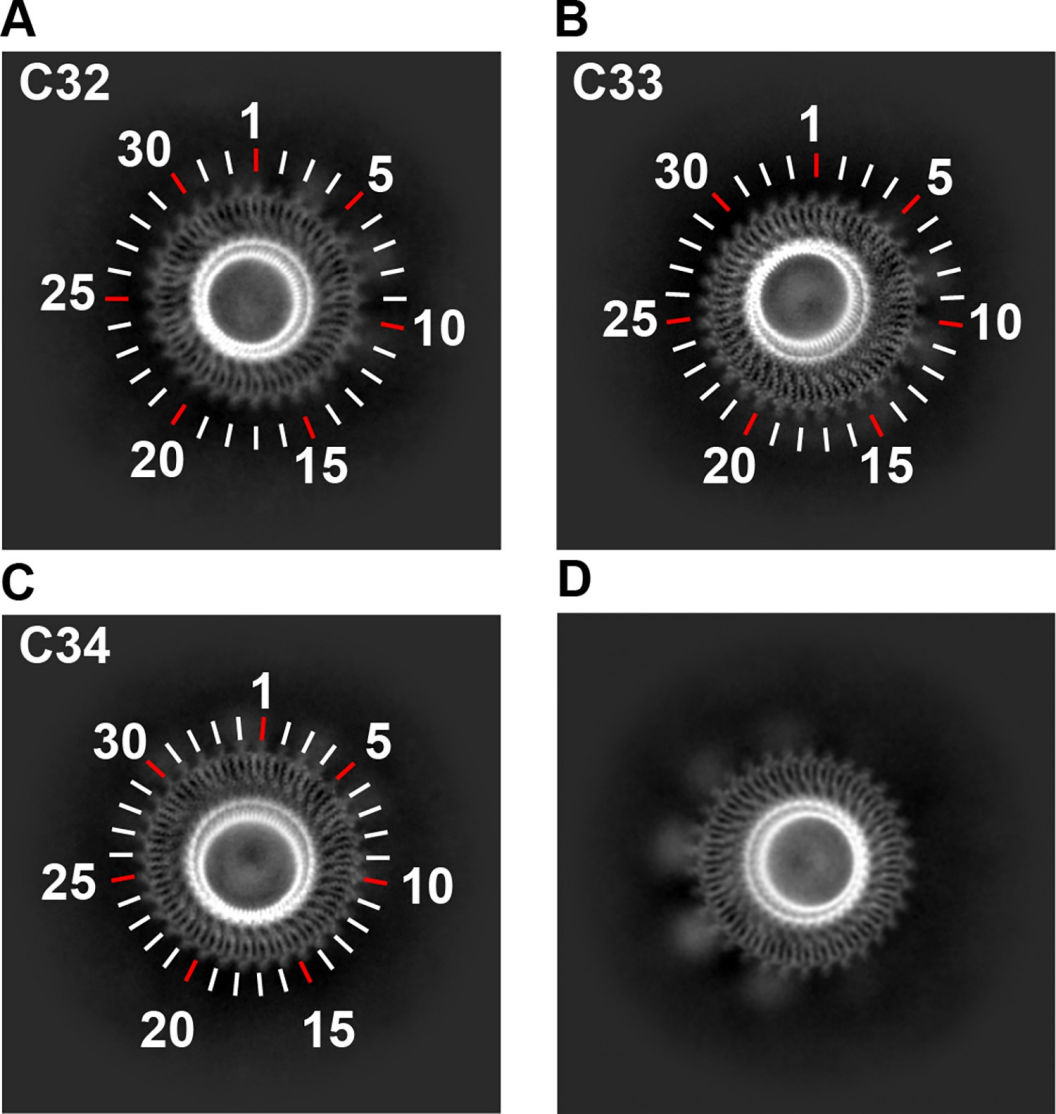
2D class averages of the MS-ring. Pre-symmetrized 2D class averages of the *en-face* views showing **(A)** C32, **(B)** C33 and **(C)** C34 symmetry. Tick marks, including colored tick marks, are included to aid in counting. **(D)** A slightly tilted view reveals globular density on the periphery and below the ring.

We next evaluated the stoichiometry of the MS-ring in 2D class averages (**S1B and S1C Fig)**. By examining the *en-face* views in the 2D class averages, where the number of subunits could clearly be counted (**Fig 3A–3C**), we could identify MS-rings that contained 32, 33, and 34 subunits. No rotational symmetry averaging is applied at this stage, indicating that this variation in ring stoichiometry is not an artifact of data handling. MS-rings containing 33 subunits represented ~80% of the population (49,944 particles). In contrast, MS-rings containing 34 subunits represented ~15% of the population (9,933 particles), and MS-rings containing 32 subunits represented only ~5% of the population (3,315 particles). This supports the hypothesis that the MS-ring can adopt more than one stoichiometry [17,36].

We used these 2D class averages to evaluate the RBMs of the MS-ring. We began with the central RBM3 domain. As with past structures, the stoichiometry of the MS-ring appears to dictate the number of observed RBM3 domains, *i*.*e*., each FliF subunit has one folded RBM3.

We next evaluated RBM2_inner_, which had a location and size similar to what was reported previously [17,19,21]. Consistent with a symmetry mismatch between RBM3 and RBM2, the stoichiometry of RBM2_inner_ was not clear in the 2D class averages, likely because the alignment was dominated by RBM3.

Below RBM2_inner_ at the base of the MS-ring complex, we observed additional density in the 2D class averages. This density suggested symmetric spacing of a large domain that did not form a continuous ring. This density was more difficult to observe in the fully *en-face* view but became prominent in 2D classes with slightly tilted views **(Fig 3D)**. The size of this density is substantially larger than would be expected for RBM1, and the location is consistent with the membrane region of the MS-ring. We propose that the transmembrane helices are located in this region.

### 3D structure of RBM3 identifies interdomain flexibility

**Fig 4:**
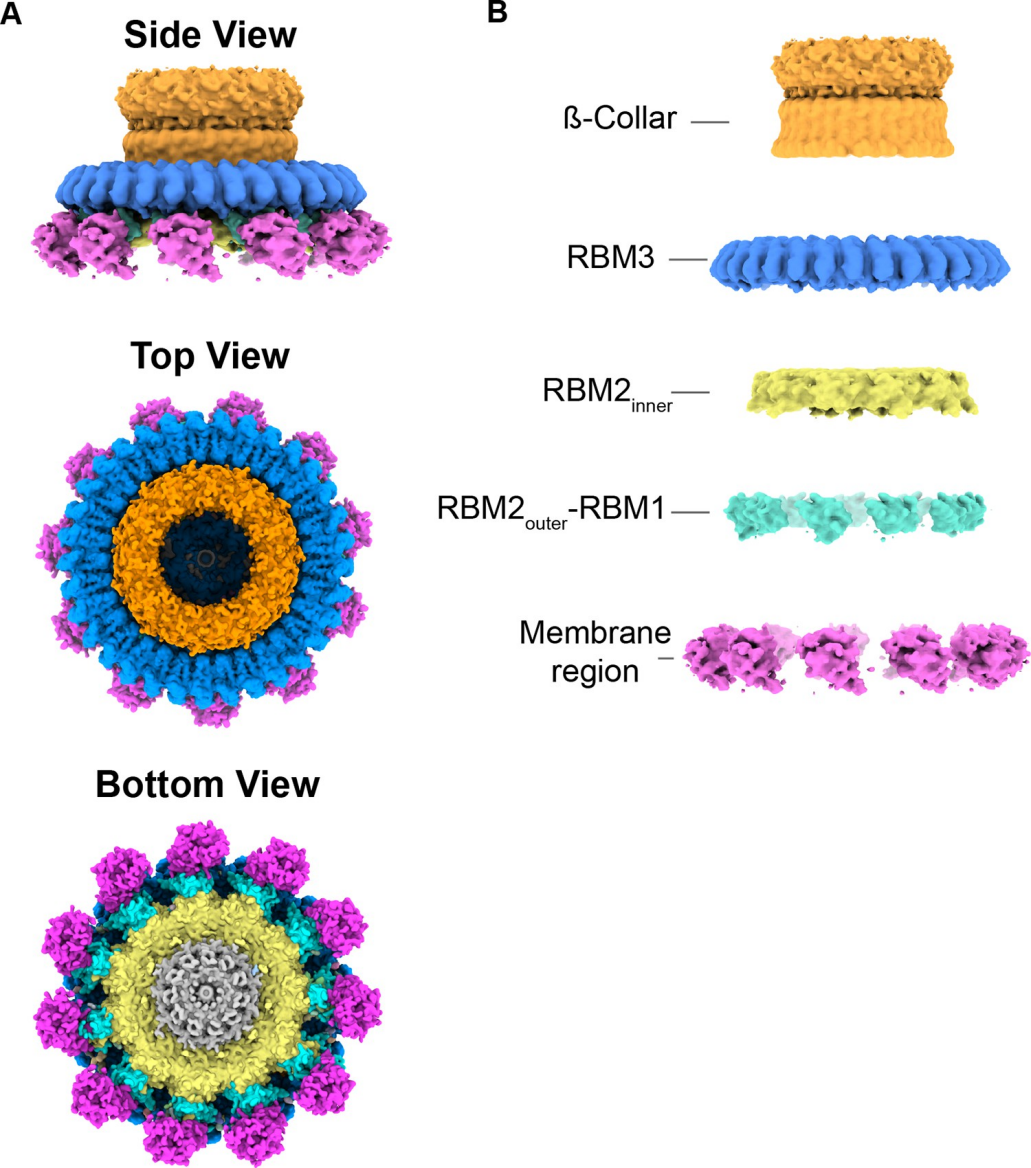
Domains of the MS-ring. **(A)** Map of MS-ring colored by domain with the ß-collar region in orange, RBM3 in blue, RBM2 in yellow, RBM2_outer_-RBM1 in cyan, and membrane region in magenta **(B)** Separation of the individual domains of the MS-ring highlights the different resolutions and symmetries of each region of the post-assembly MS-ring.

To approach the 3D structure of the MS-ring **(Fig 4A)**, we used separate symmetry averaging of the RMB2 and RBM3 domains **(S2 Fig)**. This procedure allowed high-resolution views of both RBM2 and RBM3 but resulted in the loss of connection between each domain. In addition, we could not determine whether particular stoichiometries were disproportionately biased toward a certain number of observed RBM2 domains. Following this procedure, the maps for RBM3 with C32, C33, and C34 symmetries were at resolutions of 4.2 Å, 2.9 Å, and 3.9 Å, respectively. The maps for RBM2 C21 and C22 were at resolutions of 4.4 Å and 3.8 Å, respectively.

**Fig 5:**
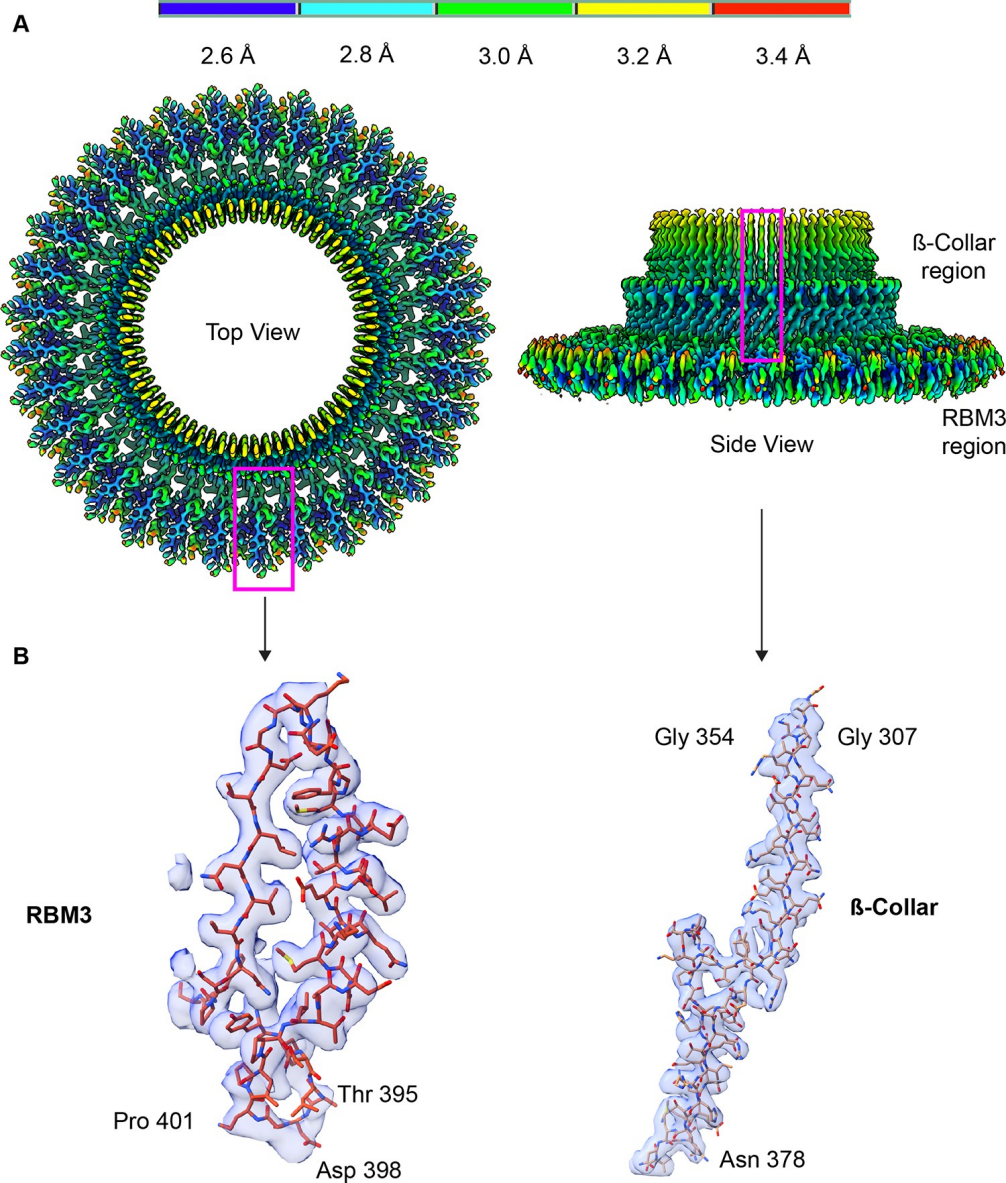
CryoEM structure of the soluble region of the MS-ring. **(A)** Map of the C33 RBM3 colored by the local resolution, which ranges from 2.6 Å in blue to 3.4 Å in red. Regions marked with the magenta boxes are shown in panel B with corresponding density. **(B)** Representative fit of models into the cryoEM density map.

**Fig 6:**
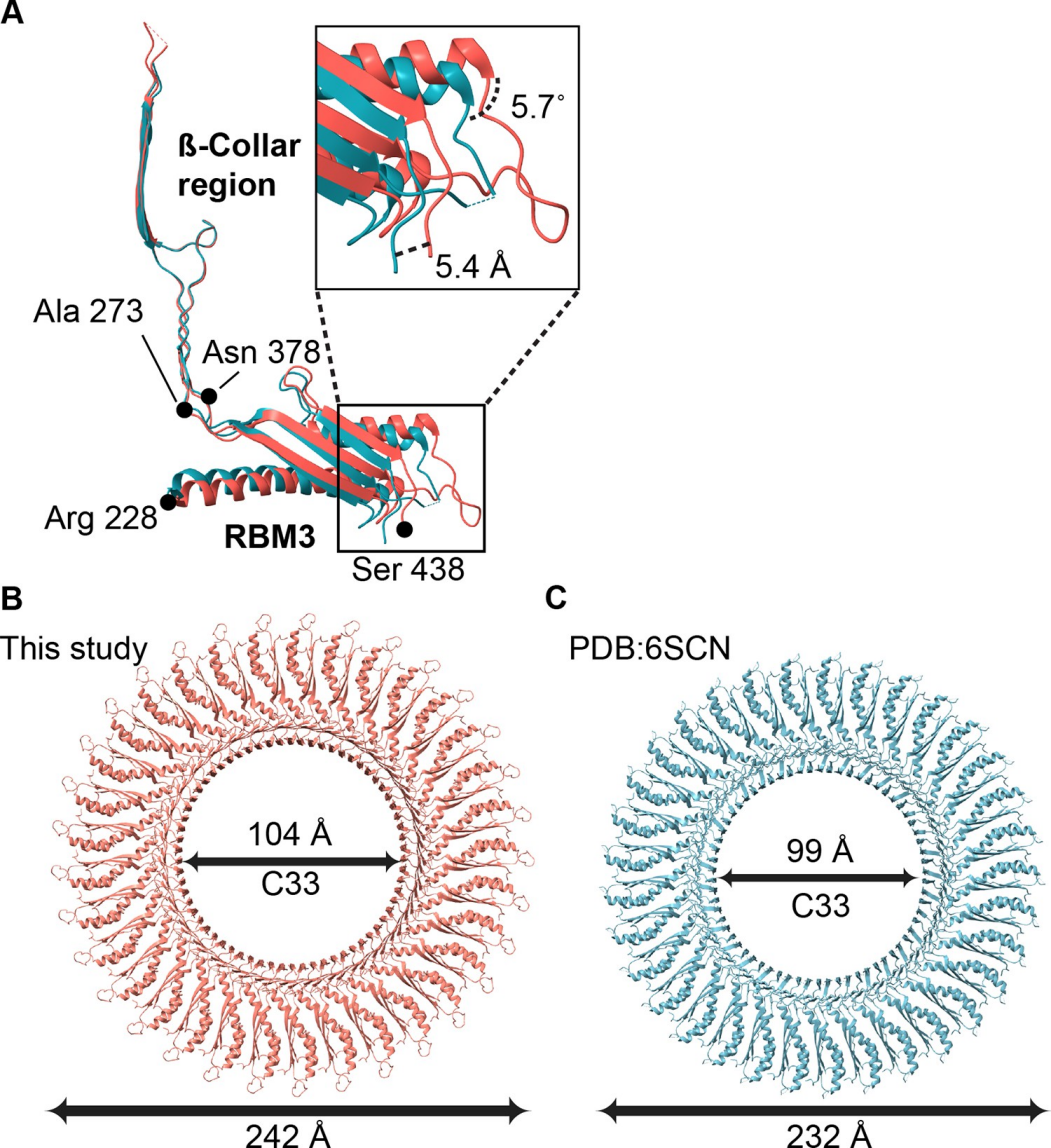
Interdomain flexibility between the β-collar and RBM3. **(A)** Superposition of the β-collar from this study (red) superimposed with the equivalent region in the coordinates deposited by Johnson *et al* (cyan, PDB:6SCN [17]) highlights a 5.7° difference in angle between the β-collar and RBM3, as calculated using DynDom [37]. This translates into a shift of position of RBM3 of 5.4 Å and a ~10 Å difference in diameter of the MS-ring, even with the same number of protomers. **(B)** The outer diameter (measured from chain A, Leu-402 to chain Q, Leu-402) and inner diameter (measured from chain A, Leu-298 to chain Q, Leu-298) of the MS-ring structure finds an outer and inner diameter of our structure of 242 Å and 104 Å, respectively. (**C**) Using equivalent residues for the measurement, the outer and inner diameter of the MS-ring structure from PDB:6SCN [17] is 232 Å and 99 Å, respectively.

The 3D map (**S2 Fig**) of the 33-mer RBM3 had an average resolution of 2.9 Å, with local resolutions between 2.6 Å and 3.4 Å (**Fig 5A**). We therefore built an atomic model into this map **(Fig 5B)**. At a global level, each RBM3 appeared to exhibit the same fold as previous models [17,20]. However, an overlay of our structure with that reported by Johnson et al [17] resulted in an RMS deviation of 1.2 Å, which is greater than would be expected for two structures of identical sequence at ~3 Å resolution. We therefore evaluated whether the RMS deviation could be artificially high because of a difference in angle between the β-collar and RBM3. As calculated by DynDom [37], there is an interdomain angle difference of 5.7˚ between our structure and that of the RBM3 structure reported by Johnson et al ([17], PDB:6SCN) **(Fig 6A)**. This difference in angle results in a difference in the diameter of the MS-ring of ~10 Å [17] **(Fig 6B and 6C)**. Separate superposition of the β collar and RBM3 to the RBM3 structure reported by Johnson et al ([17], PDB:6SCN) results in a lower RMS deviation, 0.77 Å and 0.58 Å, respectively. As a minor note, residues 395–401 of RBM3 in the 33-mer were associated with clear density **(Fig 5B)** but were not included in the previously published models [17].

For the 32-mer and 34-mer **(S4 Fig)** MS-ring, we saw a difference in the diameter of ~10 Å when compared to previously deposited structures with the same stoichiometry (C32-mer: EMD-10560, C34-mer: EMD-10147) [17]. This was similar to the difference in the diameter of C33-mer when compared to the previous C33 structure (PDB:6SCN [17]) **(Fig 6B and 6C)**.

### 3D structure of the lower region of the MS-ring shows RBM2_inner_ and RBM2_outer_-RBM1

**Fig 7:**
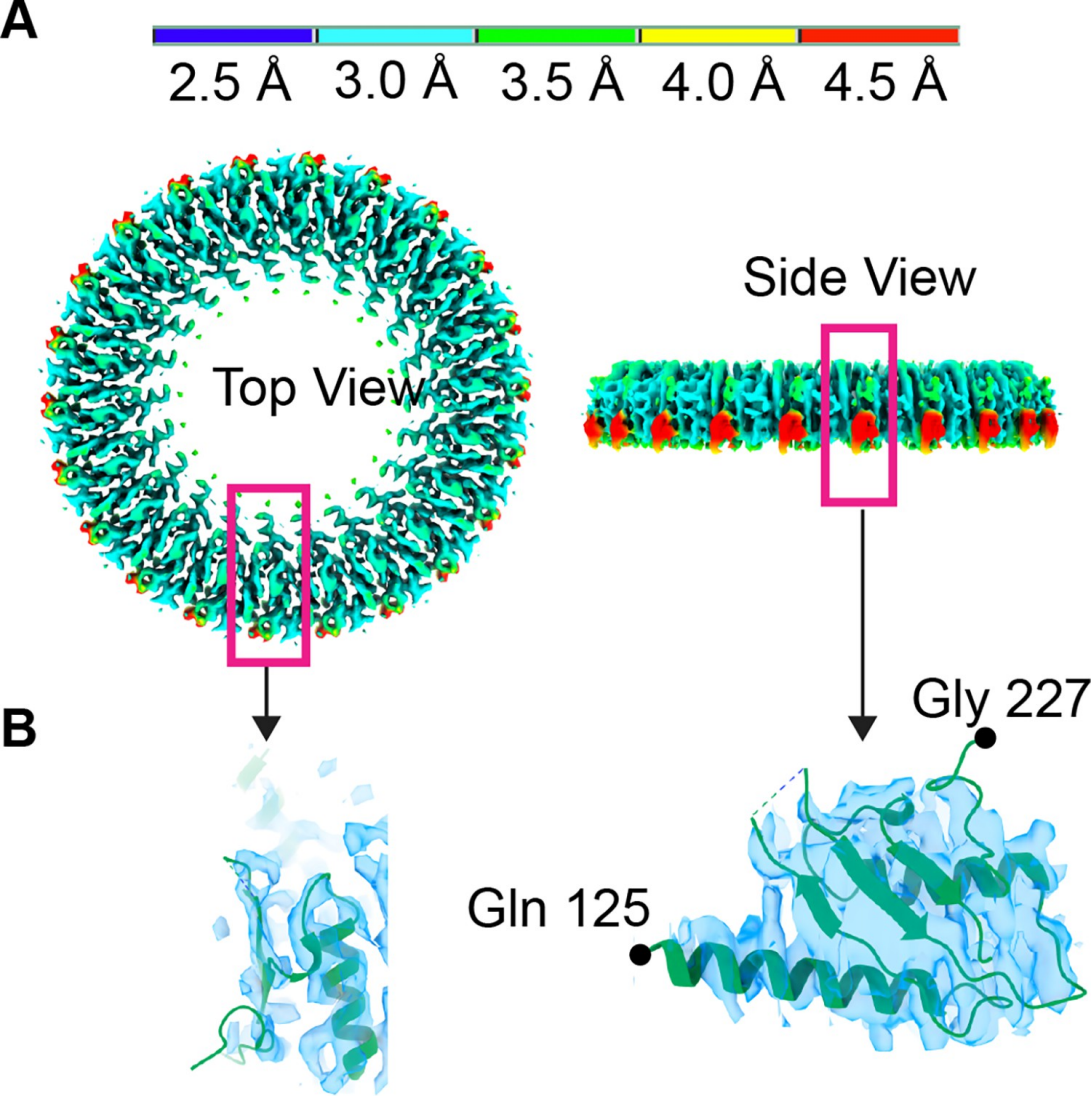
CryoEM structure of the RBM2_inner_ ring of the MS-ring. **(A)** Map of the C22 RBM2_inner_ colored by the local resolution, which ranges from 2.5 Å in blue to 4.5 Å in red. Regions marked with the magenta boxes are shown in panel B. **(B)** Two views of the RBM2 protomer in the RBM2_inner_ position superimposed onto the cryoEM map. The density begins at Gln-125 and ends at Gly-227.

Past structures of the MS-ring showed heterogeneity of the stoichiometry and position in RBM2, with a consensus that this is biologically relevant [17,19,20]. The maps associated with this post-assembly MS-ring identify only minor variations of what has been reported. Specifically, past work showed that RBM2 could occupy distinct positions [17,20]. One of these positions was in a ring located below RBM3 and was termed RBM2_inner_ [17,20] **(Figs 4B and 7A)**. The second position was in discontinuous globular density on the periphery of RBM2 which was interpreted as RBM2 that was tightly-associated with RBM1 [20] and is termed RBM2_outer_-RBM1 (**Fig 4B)**.

**Fig 8:**
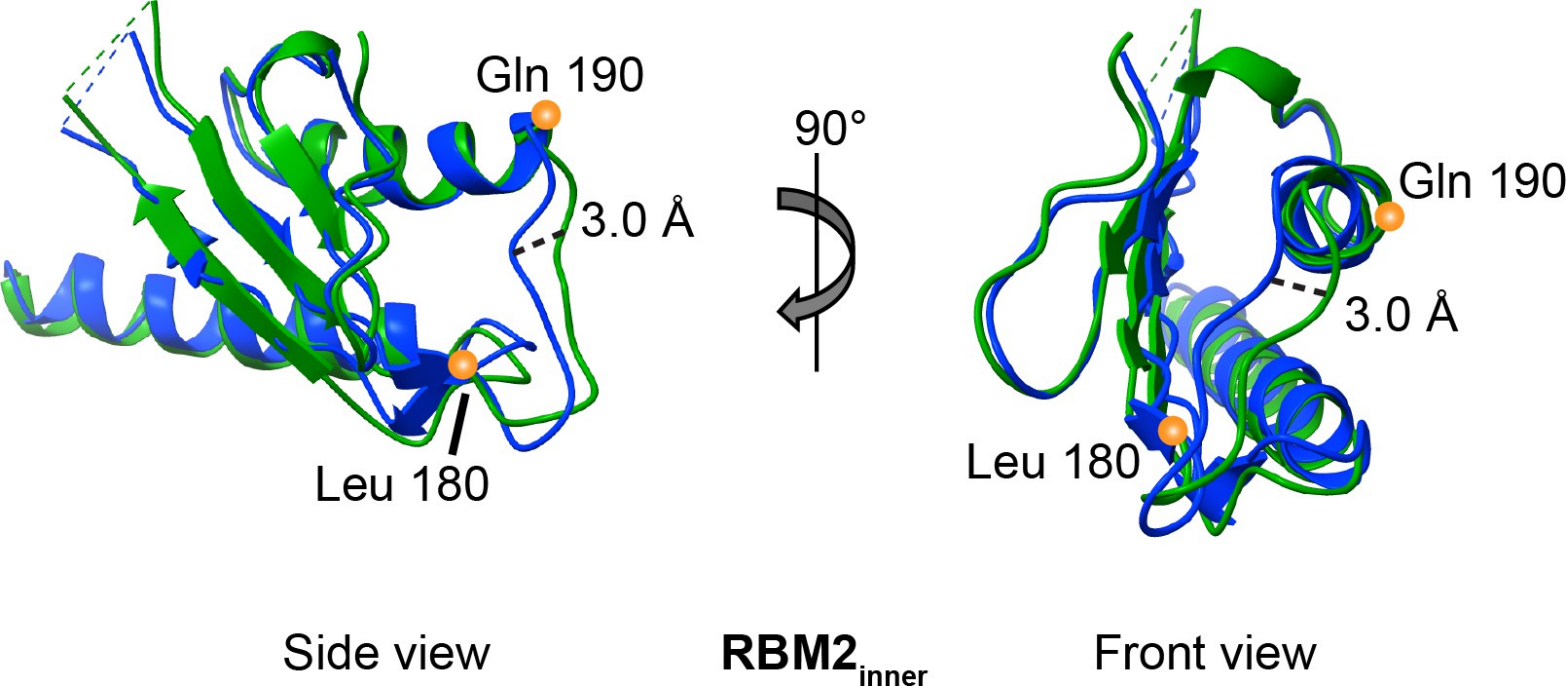
Superposition of RBM2_inner_ shows domain flexibility. Orthogonal views of the 22-mer RBM2_inner_ model from this study (green) superimposed with the 22-mer RBM2_inner_ deposited by Johnson *et al* (blue, PDB:6SD5 [17]). Differences in the Cα positions are observed throughout the models rather than localized to a specific region. These are not influenced by an interdomain angle change. The maximal shift in position is 3.0 Å.

As in previous reports [17,20], we observe more than one stoichiometry for the RBM2_inner_ ring; here, we observe 21- and 22-mers. The resolutions of these RBM2_inner_ densities are 4.1 Å and 3.7 Å, respectively. We built a model for the 22-mer RBM2_inner_ (**Fig 7B**) which encompasses Gln-125 to Gly-227 and we compared this to the deposited 22-mer structure (PDB:6SD5, [17]). Although our model for RBM2_inner_ appeared similar to the previous structure at a global level [17], the RMS deviation was 1.2 Å for C_α_ atoms, which is higher than anticipated at this resolution. The main positional differences were found in a long loop (residues Leu-180 to Gln-190) between secondary structural elements and at the termini **(Fig 8)**. However, the overlay had a high RMS deviation throughout. One possibility is this high RMS deviation reflects the ability of RBM2 to adjust to different MS-ring diameters and symmetries.

**Fig 9:**
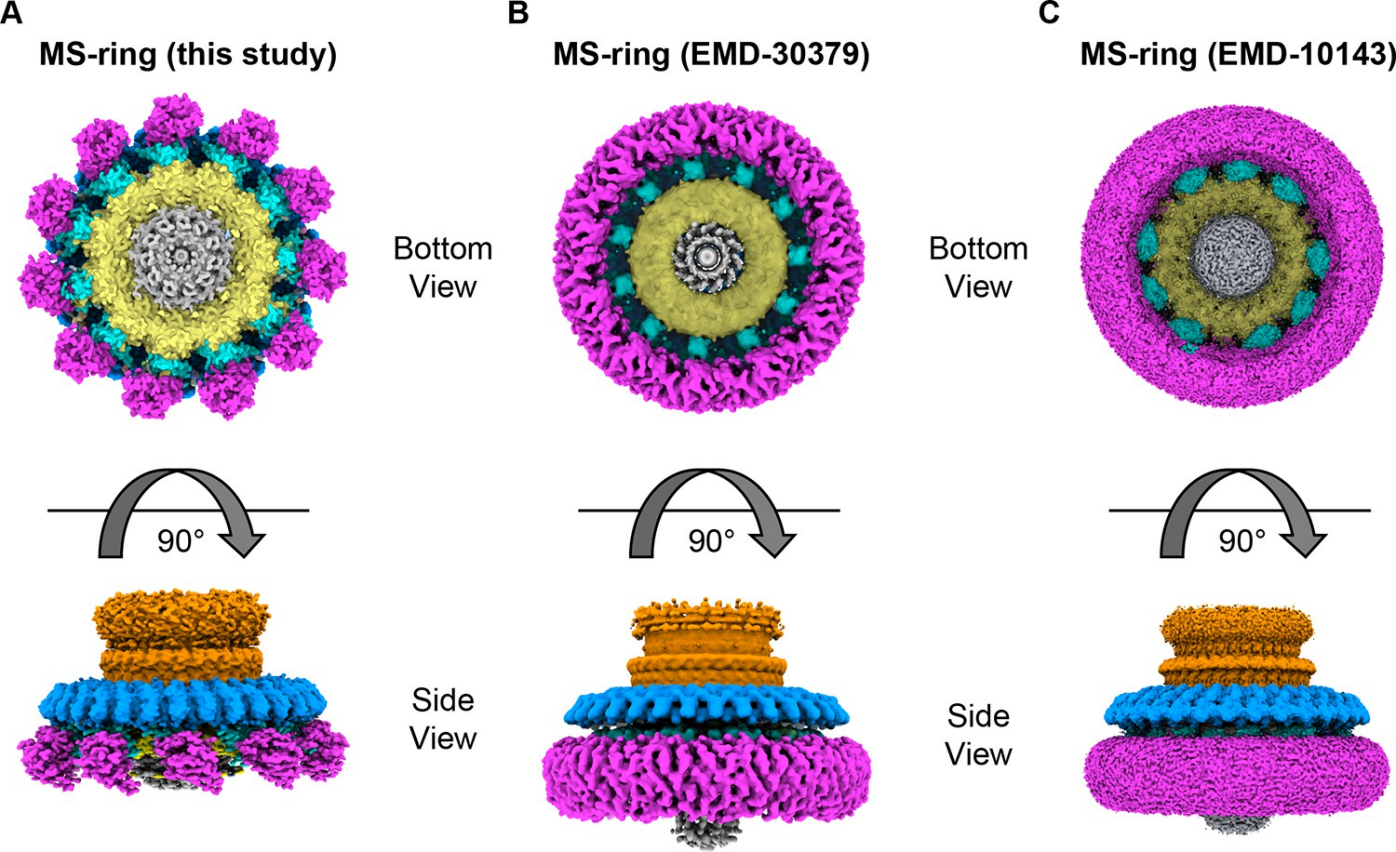
Comparison of available high-resolution cryoEM density maps [17,20]. In each figure panel, RBM3 is shown in blue, the β-collar is shown in orange, RBM2_inner_ is shown in yellow, RBM2_outer_-RBM1 is shown in cyan, and the transmembrane domains are shown in magenta. **(A)** EM density map of the MS-ring reported here **(B)** EM map for MS-ring from EMD-30379 [20] **(C)** EM map for MS-ring from EMD-10143 [17].

We also observed additional densities on the periphery of RBM2_inner_ ring **(Fig 9A)**. These densities have previously been reported as either 11 or 9 copies of RBM2_outer_-RBM1 [17,20] **(Fig 9B and 9C)**. We observe 11 regions of density (**Fig 9A**), which we tentatively assign as an RBM2_outer_-RBM1 complex.

### An 11-fold symmetric globular density below RBM3

On the periphery of the MS-ring, below RBM3, we observe additional density in both the 2D classes **(Fig 3D)** and in the 3D reconstruction **(S4 Fig)**. While this density is positioned in the location that is classically assigned as the membrane region, it forms separated regions with 11-fold symmetry and does not form a contiguous ring as shown in the past [9,17,20] (**Fig 9B and 9C**). Although we were not able to improve the density enough to trace a model with confidence, the size of this density is consistent with the size of transmembrane helices within an LDAO micelle. The correlation of these densities with the stabilization of RBM3 residues 395–401 potentially suggests an architecture with discrete membrane-spanning domains.

## Discussion

A central part of the bacterial flagellar motor is the MS-ring, which is composed entirely of multiple copies of the FliF subunit. Major functions of this ring are to support the assembly of the switch and to stabilize the assembled motor. Past structures showed variation in stoichiometry, domain positioning, and organization in the RBM3, RBM2, and RBM1 of the FliF (**Fig 9B and 9C**). While there is broad consensus that RBM2 could adopt multiple stoichiometries, positions, and orientations, there remains major debate as to whether the overall number of FliF subunits can vary, with some reports suggesting that the variation was due to partial proteolysis of the sample or misaligned particles. Here, we observe clear 2D class averages of RBM3 with C32, C33, and C34 symmetry **(Fig 3A–3C)**, but did not observe any proteolysis in the sample (**S1A Fig**). This supports variation in the stoichiometry. We suggest that the differences in stoichiometry could allow for stepwise increases in torque when responding to increases in load, as has been reported [25]. Given the nature of this motor, these stoichiometry differences might easily be explained by small differences in growth conditions during expression.

We were able to develop high-quality maps of regions of the MS-ring and assign these to regions of the FliF sequence (**Figs 5 and 7**). Consistent with past reports, our MS-ring structure contains different numbers of folded RBMs in each FliF subunit. We observe 32–34 RBM3s, 21–22 RBM2_inner_, 11 RBM2_outer_-RBM1 complexes, and 11 putative membrane-spanning domains. One potential combination of these folded domains results in a symmetry match (33 RBM3; 22 RBM2_inner_; 11 RBM2_outer_; 11 RBM1 and 11 membrane-spanning), while the remaining combinations of domains result in a symmetry mismatch. There has been speculation on the role of symmetry mismatch in the optimal function of biological motors, with one theory being that the symmetry mismatch prevents the formation of energy minimum for a single position [38]. Such an energy minimum could decrease the rate of rotation, potentially by preferentially trapping one state. In support of a role of symmetry mismatch in optimal motor function, symmetry mismatch is observed in other biological motors. This includes both rotary motors such as the F_1_F_o_ ATP synthase [39–41] and non-rotary motors, such as sarcomere filaments and the titin protein [42].

A comparison of the MS-ring in this study to that in past reports [17,20] **(Fig 9)** shows that in addition to the variability in the number of subunits and domains **(Figs 3, 4B and S4)**, there is a difference in the interdomain angle between the β-collar and RBM3 **(Fig 6A)** as well as structural plasticity in RBM2 (**Fig 8)**. All the above findings affect the diameter of the MS-ring **(Fig 6B and 6C)**. While speculative, the ability to change the diameter could affect the torque generated.

In general, the flexibility that is observed upon the comparison of multiple structures of the MS-ring could have functional impacts. For example, flexibility could be important to accommodate the spinning flagellum. Flexibility could also allow for different numbers of subunits to be assembled into the switch complex or the entire flagellum **(S4 Fig)**.

In considering how flexibility might impact flagellar assembly, one of the functions of the MS-ring is as a template for assembly for other regions of the flagellar rotor, including the switch complex. The change in MS-ring diameter could support more than one stoichiometry in the larger switch complex, further extending the variation in symmetry to the next level of this motor. In more careful consideration of assembly, it is tempting to focus on the regions of FliF that are physically adjacent to the switch complex or that have major structural differences between the pre- and post-assembled states. One region that this calls attention to is the transmembrane helices, which are located at the N- and C-termini in the sequence **(Fig 1B)**. While deletion of the membrane-spanning region itself has not been reported, deletion of the N-terminal region leading into the first transmembrane helix reduces flagellar assembly [43]. Our post-assembly structure **(Fig 9A)** shows that these membrane-spanning helices form discrete domains at the base of the MS-ring. In contrast, the pre-assembly structures [9,17,19,20,22,26] show a contiguous ring **(Fig 9B and 9C)**. It is not yet clear which of these possibilities represents the true architecture of the membrane region of the MS-ring. For example, it could be envisioned that the membrane-spanning regions do not form helical bundles until after association with the switch complex. Alternatively, it could be envisioned that a contiguous ring [9,17,19,20,22,26] is an artifact of data handling, or an artifact of the detergent breaking up the normal helix packing [44,45]. Finally, it could be envisioned that the 11-fold structures are an artifact of the LDAO detergent. We believe that the discrete densities are less likely to be an artifact because a more ordered state is more likely to be biologically relevant. A structure where the membrane-spanning helices can be unambiguously assigned will be necessary to distinguish between these possibilities.

Taken together, the structure here and comparisons to previously available structures of the *S*. *enterica* serovar *typhimurium* MS-ring [9,7,19–22,26] indicate structural plasticity at many levels. Differences are observed in the number of FliF subunits that form the MS-ring, the percentage of each FliF subunit that folds into domains versus remains disordered, the structure of RBM2, the angles between the β-collar and RBM3, the observation of 11 domains for RBM2_outer_-RBM1, and the appearance of the density putatively assigned as the membrane-spanning region. Much of this plasticity is consistent with a biological function that may require an ability to react to changes in the environment. The changes in the organization and position of the membrane-spanning region may also suggest that domain movements accompany the assembly or the disassembly of the MS-ring.

## Supporting information

S1 Fig Sample preparation and 2D classification. **(A)** SDS PAGE gel showing purified full length FliF (61 kDa) following separation from the FliGMN switch. **(B)** Schematic of the data processing workflow from data collection through 2D class averages. Beginning with 11,627 movies, we divided these into two groups, termed group “A” and “B”. Following CTF estimation, we removed micrographs with poor ice. Group “A” contained the un-tilted micrographs (5,597 movies) while group “B” contained all 10,707 micrographs. **(C)** Representative 2D class averages of the MS-ring. The scale bar is 100 Å.
(TIF)

S2 Fig Workflow for structure determination with C33 and C11 symmetry. Representative 3D reconstructions using only data from group “A” micrographs are shown at relevant points in the workflow. Here, C33 symmetrization shows the RBM3 and β-collar, while C11 shows all domains. The GS-FSC indicates that the high-resolution map of C33 RBM3 is at 2.9 Å resolution.
(TIF)

S3 Fig Workflow for regions of the MS-ring with other symmetries: C32, C34, C21, and C22. Summary of the workflow used to obtain the 3D maps for the C32 RBM3 ring, the C34 RBM3 ring, the C21 RBM2_inner_ ring, and the C22 RBM2_inner_ ring. These maps used all micrographs, i.e. group “B”. Representative models are shown at relevant points in the workflow.
(TIF)

S4 Fig Different oligomeric states of RBM3 and RBM2_inner_. Figure shows the top view of C32- (brown), C33- (blue) and C34-mer (violet) RBM3 and bottom view of C21- and C22-mer RBM2_inner_ along with their inner and outer diameter.
(TIF)
