# Supplementary figures and images for: CryoEM structure of a post-assembly MS-ring reveals plasticity in stoichiometry and conformation
Source: PLoS One. 2023 May 19;18(5):e0285343. doi: 10.1371/journal.pone.0285343 (PMC10198558; doi:10.1371/journal.pone.0285343)

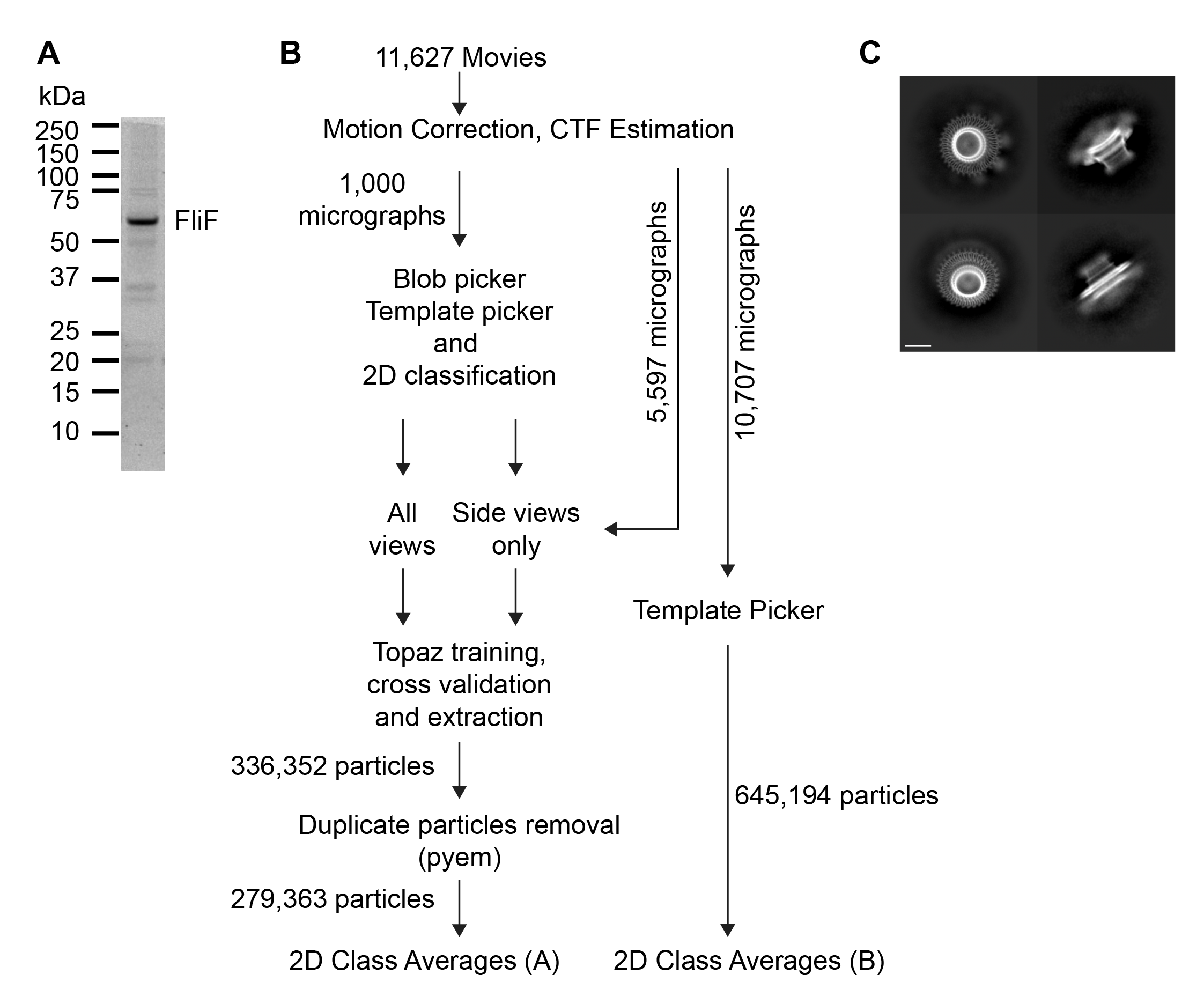

Supplement: S1 Fig — (A) SDS PAGE gel showing purified full length FliF (61 kDa) following separation from the FliGMN switch. (B) Schematic of the data processing workflow from data collection through 2D class averages. Beginning with 11,627 movies, we divided these into two groups, termed group “A” and “B”. Following CTF estimation, we removed micrographs with poor ice. Group “A” contained the un-tilted micrographs (5,597 movies) while group “B” contained all 10,707 micrographs. (C) Representative 2D class averages of the MS-ring. The scale bar is 100 Å. (TIF) [file pone.0285343.s001.tif]

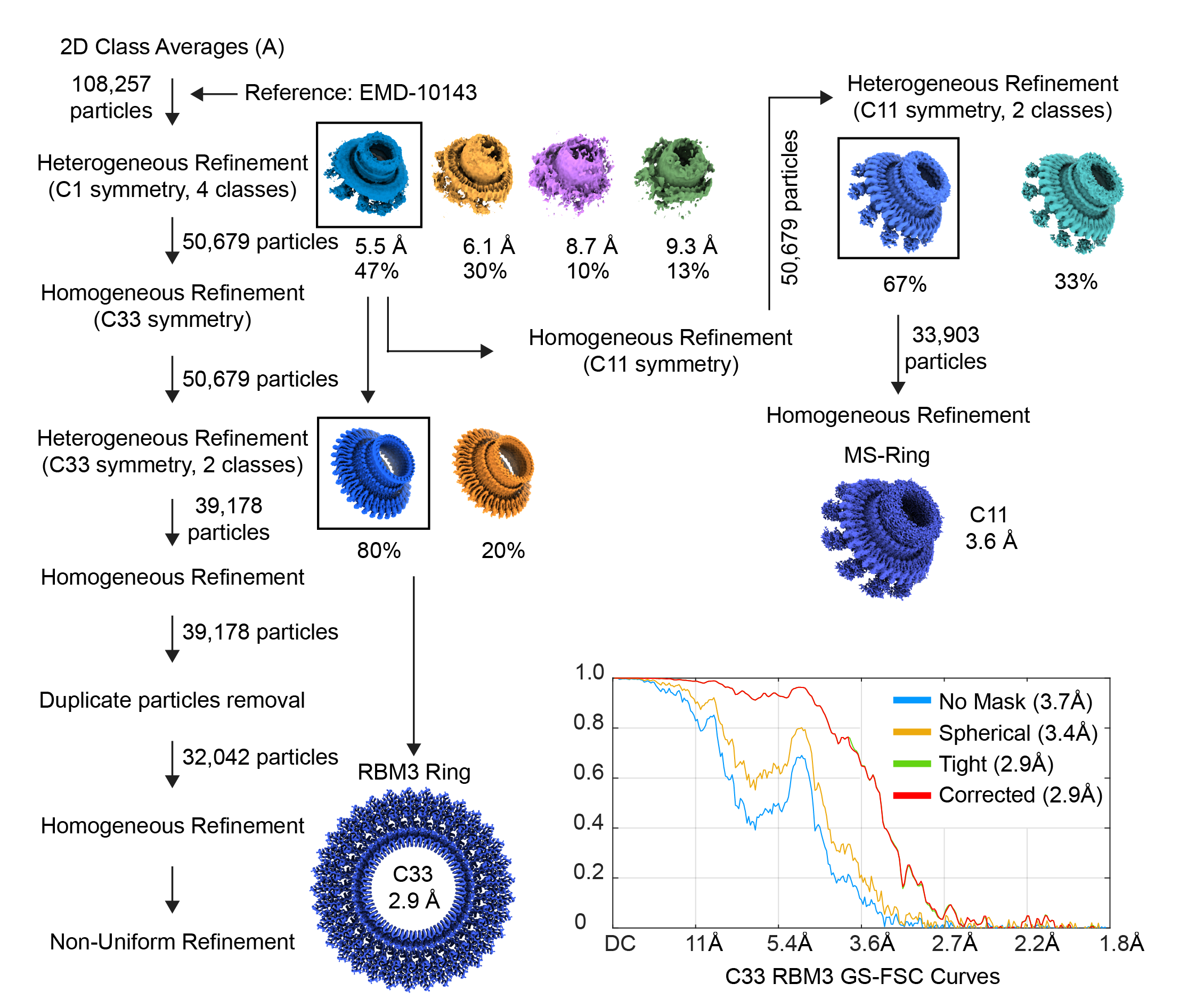

Supplement: S2 Fig — Representative 3D reconstructions using only data from group “A” micrographs are shown at relevant points in the workflow. Here, C33 symmetrization shows the RBM3 and β-collar, while C11 shows all domains. The GS-FSC indicates that the high-resolution map of C33 RBM3 is at 2.9 Å resolution. (TIF) [file pone.0285343.s002.tif]

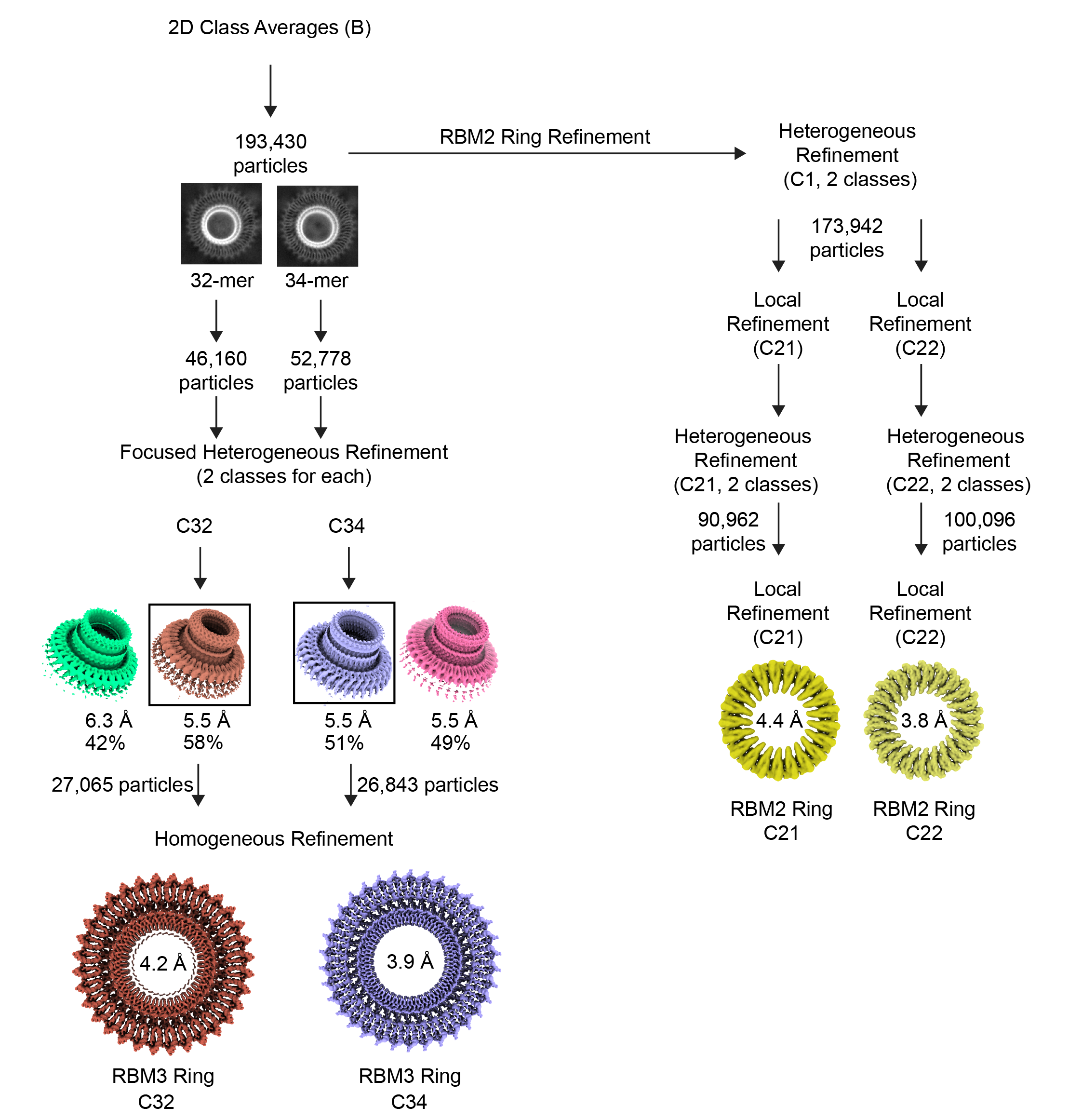

Supplement: S3 Fig — Summary of the workflow used to obtain the 3D maps for the C32 RBM3 ring, the C34 RBM3 ring, the C21 RBM2inner ring, and the C22 RBM2inner ring. These maps used all micrographs, i.e. group “B”. Representative models are shown at relevant points in the workflow. (TIF) [file pone.0285343.s003.tif]

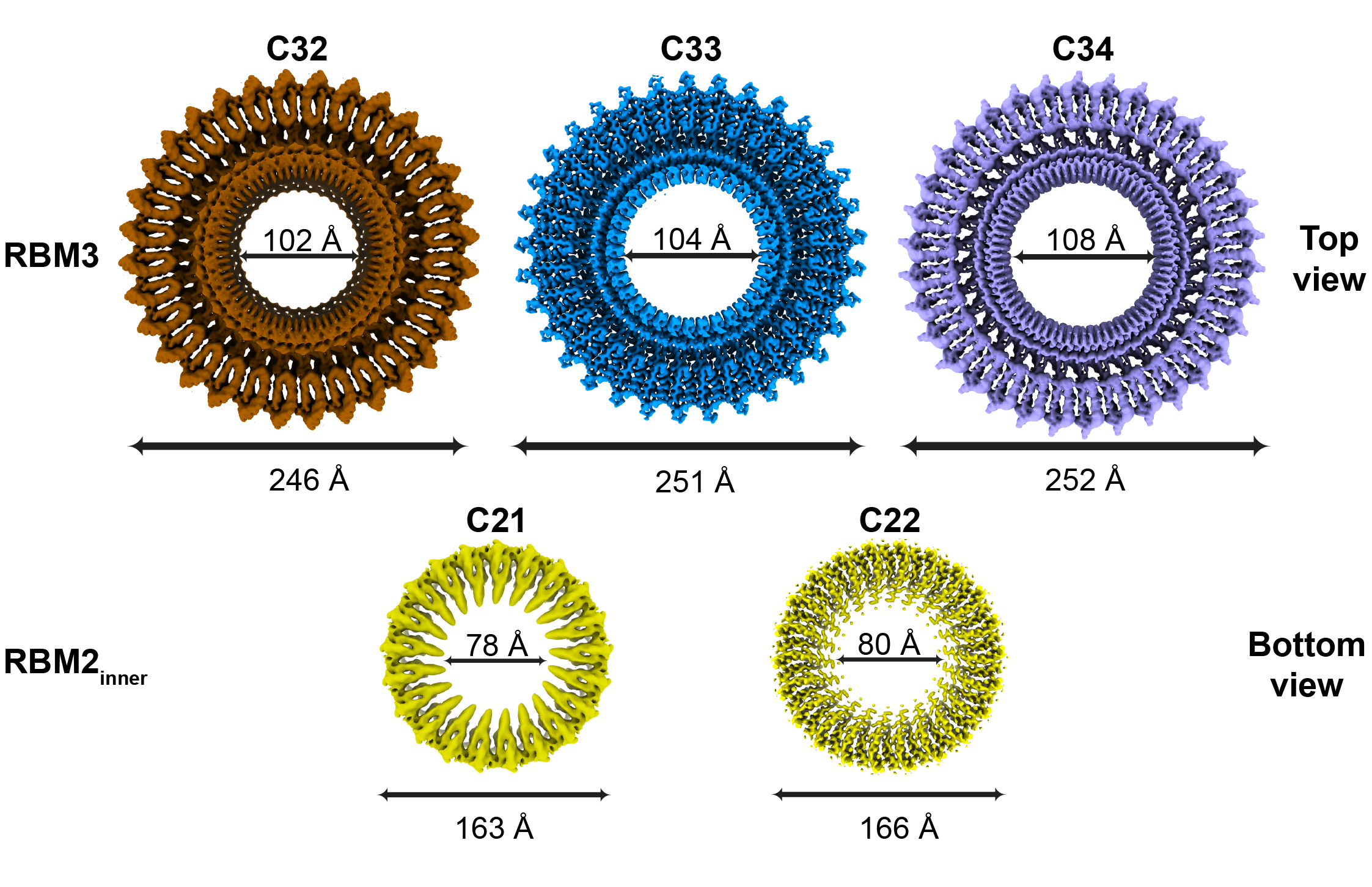

Supplement: S4 Fig — Figure shows the top view of C32- (brown), C33- (blue) and C34-mer (violet) RBM3 and bottom view of C21- and C22-mer RBM2inner along with their inner and outer diameter. (TIF) [file pone.0285343.s004.tif]
